# The complete mitogenome and phylogeny analysis of *Pareuchiloglanis longicauda* (Yue, 981) (Siluriformes: Sisoridae)

**DOI:** 10.1080/23802359.2021.2002203

**Published:** 2021-11-26

**Authors:** Wei Dai, Zhihong Yin, Nannan Li, Shuifeng Ye

**Affiliations:** School of Life Sciences, Shangrao Normal University, Shangrao, China

**Keywords:** *Pareuchiloglanis longicauda*, mitochondrial genome, phylogenetic analysis

## Abstract

*Pareuchiloglanis longicauda*, a Sisorid fish that is distributed in the upper Pearl River. In this study, the complete mitogenome of *P. longicauda* was sequenced using traditional Sanger sequencing approach. The 16,588 bp genome was consisted of 2 rRNAs, 22tRNAs, 13 protein-coding genes (PCGs) and 1 control region. The 13 PCGs started with a traditional ATG and end with stop codon TAA, TAG, TGA, TA or a single T base. Phylogenetic analysis based on 13 PCGs from 22 species using maximum-likelihood method produced three major clades (Clade I, II and III). Unexpectedly, our mitogenome exhibited only 92.12% identity to the previously published one (GenBank accession no. KP872693) with differences mainly located in the gene region. Furthermore, *Pareuchiloglanis* did not form a monophyletic genus and *P. longicauda* had the closest relationship with *P. macrotrema*. The result suggested that more complete mitogenomes are needed to reveal the phylogenetic placement of *Pareuchiloglanis* in the family Sisoridae.

*Pareuchiloglanis longicauda* is an endemic Sisorid fish in the upper Pearl River drainage, which is the largest river in southern China (Yue 1981). Most previous research on *P*. *longicauda* has focused on its morphological characteristics (He 1996; Dao et al. 2020). However, controversy mainly occurs in the monophyly of *Pareuchiloglanis* (Ma et al. 2015; Lv et al. 2018). The genus was considered being monophyletic only when they were assigned to *Pseudexostoma* and *Oreoglanis* were included according to morphological characters (He 1996; Thomson and Page 2006). Recent research argued that there are two groups for the genus: the large-gill-opening group and the small-gill-opening group, and *P. longicauda* belongs to the former one (Dao et al. 2020). Here we sequenced and characterized the complete mitochondrial genome for *P. longicauda* from the upper Pearl River and tried to investigate phylogenetic placement of this species in relation to other Sisorid fish. The voucher specimen was sampled from the upper Pearl River (25.13897 N, 04.95406 E), Xingyi city, Guizhou Province, China in August, 2020 and stored in the fish collection of Shangrao Normal University, Shangrao, China (Voucher no. MLR20200811). A small portion of muscle tissue was clipped from the specimen and total genomic DNA was extracted using the DNeasy tissue kit (Qiagen) according to the manufacturer’s instructions. Four overlapping primers were used to amplify the complete mtDNA from the genomic DNA (Ma et al. 2015). Subsequently, the sequencing of PCR products was carried out based on Sanger method. Raw fragment reads were assembled to generate a complete mitochondrial genome sequence. Possible PCGs encoded by the genome were predicted using Geneious^®^ 11.1.5 software (Kearse et al. 2012). MITOS was used for annotating rRNA genes and tRNA genes (Bernt et al. 2013). Further, 22 Sisorid mitogenomes were obtained from GenBank and then a matrix including 22 species were aligned by MUSLE (Edgar 2004). Finally, we retrieved 13 PCGs from the aligned matrix to construct a phylogenetic tree using MEGA X with a mtREV24 + G + I model and 1000 replicates as bootstrap parameter set (Kumar et al. 2018).

The complete mitogenome of *P. longicauda* (GenBank nos: MZ618235) was found to be a 16,588 base pairs (bp) circle with the GC content as 43.76%, which encoded the typical 13 PCGs (ND1, ND2, ND3, ND4, ND4L, ND5, ND6, COI, COII, COIII, ATP6, ATP8 and Cyt b), 2 ribosomal RNAs (12S and 16S rRNA), 22 tRNAs, and 1 D-loop. ND6 was the only PCG encoded on the L-strand of the genome. ATG was the initiation codon for all 13 PCGs. Five types of stop codons were detected: TAA (ND1, ATP8, ATP6, ND4L and ND5), TAG (ND2, COX1 and ND6), TGA (ND4), TA- (COX3) and T- (COX2, ND3 and CYTB) (Supplementary Table 1). It should be worth noting that we found 1,295 different nucleotide sites between our mitogenome and the one published previously (GenBank nos: KP872693) (Supplementary Table 2), among which 1,098 nucleotide sites were in the gene region (Ma et al. 2015).

To the best of our knowledge, the mitogenome of *Pareuchiloglanis myzostoma* (GenBank nos: MK617319) was first subject to a phylogeny inference in this study. In our maximum likelihood topology, most nodes obtained high supported values (Figure 1). Twenty-two species were divided into three major well-supported clades: Clade I (Sisorini), Clade II (Glyptothoracini) and Clade III (Glyptosternini + Pseudecheneidini). The result denied the monophyly of the genus *Pareuchiloglanis*, which was supported by previous mitogenomes studies (Ma et al. 2015; Lv et al. 2018). Moreover, *P. longicauda* had a closer relationship with *P. macrotrema* than other four species of *Pareuchiloglanis*, whereas the closest relationship between *P. longicauda* and *Creteuchiloglanis* had been reported recently (Ma et al. 2020). To date, a total of 16 valid species (*P. gongshanensis* and *P. kamengensis* were assigned to *Creteuchiloglanis* according to GenBank) of *Pareuchiloglanis* had been recognized (Thomson and Page 2006) with only 6 mitogenomes of *Pareuchiloglanis* available. Anyway, phylogenetic relationships using different Sisorid fish produced conflicting results. (Ma et al. 2015; Sonnack et al. 2018; Cui et al. 2019; Ma et al. 2020). Our study suggested that more complete mitogenomes are needed to make the phylogenetic position of the genus *Pareuchiloglanis* in the family Sisoridae clear.

**Figure 1.**
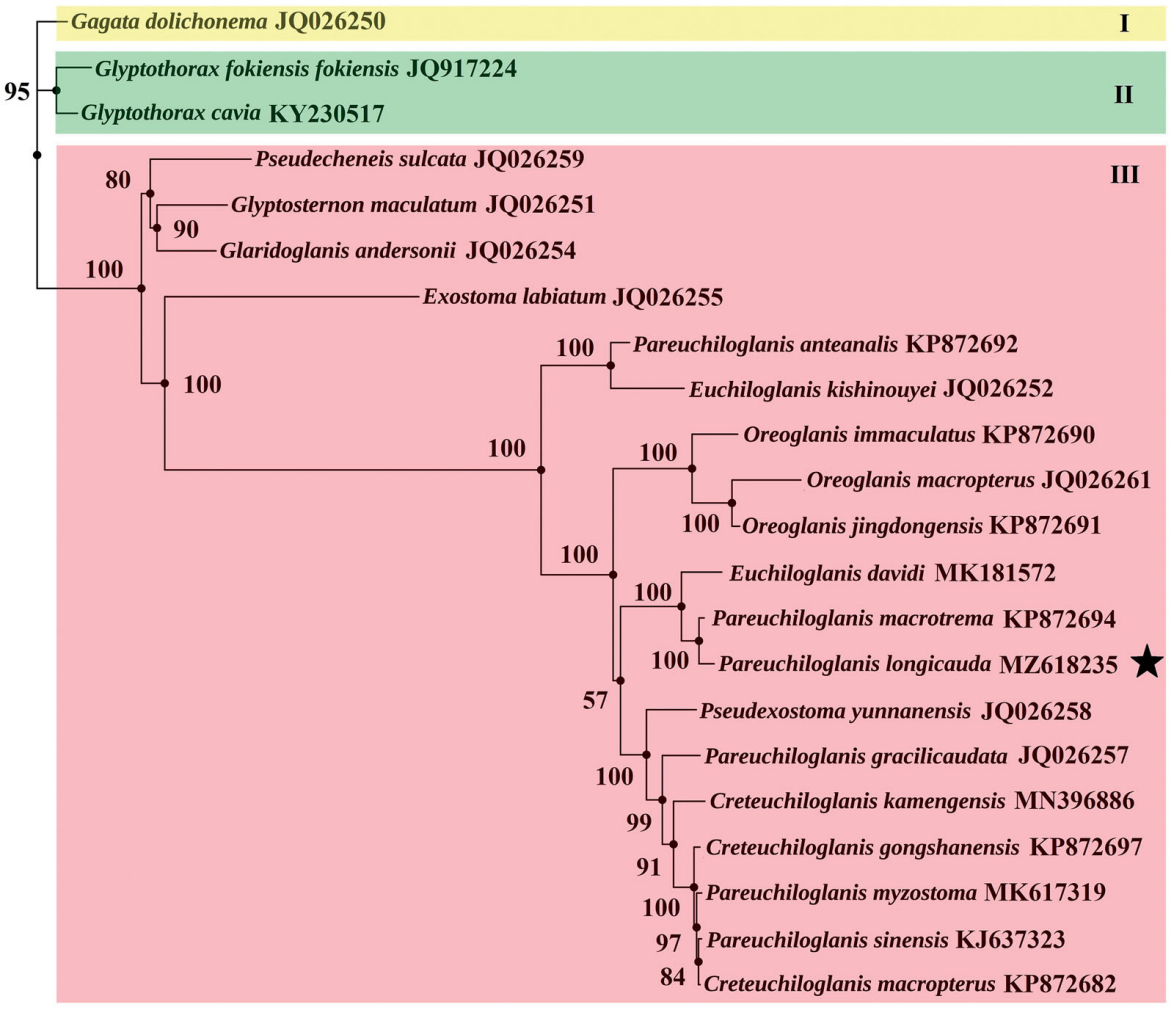
Maximum likelihood tree based on 13 PCGs of 22 Sisorid mitogenomes. Bootstrap support values are shown on branches. The species names are followed by GenBank codes. “★” indicates the position of *P. longicauda* in this study. *Pseudecheneis sulcata* represents the only species of Pseudecheneidini.

## Data Availability

Data supporting the study findings are available in GenBank of NCBI at http://www.ncbi.nlm.nih.gov, reference number MZ618235. The associated BioProject and Bio-Sample numbers are PRJNA748463 and SAMN20335858, respectively.
